# The extracellular vesicles of boar seminal plasma contain oxytocin at levels associated with fertility

**DOI:** 10.20517/evcna.2025.94

**Published:** 2025-12-16

**Authors:** Ana Parra, Pablo Martínez-Díaz, María Botía, Marina López-Arjona, Xiomara Lucas, Isabel Barranco, Jordi Roca

**Affiliations:** ^1^Department of Medicine and Animal Surgery, Faculty of Veterinary Science, University of Murcia, Murcia 30100, Spain.; ^2^International Excellence Campus for Higher Education and Research “Campus Mare Nostrum”, Institute for Biomedical Research of Murcia (IMIB-Arrixaca), University of Murcia, Murcia 30003, Spain.; ^3^Interdisciplinary Laboratory of Clinical Analysis, Interlab-UMU, Regional Campus of International Excellence Campus Mare Nostrum, University of Murcia, Murcia 30100, Spain.

**Keywords:** Extracellular vesicles, fertility, oxytocin, porcine, seminal plasma

## Abstract

**Aim:** Pig seminal plasma (SP) contains oxytocin (OXT) at levels associated with the fertility outcomes of boars used for artificial insemination (AI). However, OXT easily volatilizes when circulating freely, making it difficult to maintain stability in AI seminal doses. The hypothesis is that OXT is stably carried in seminal extracellular vesicles (EVs). This study aimed to determine the following: (1) whether seminal EVs carry OXT and, if so, where they carry it; (2) the source of seminal EVs carrying OXT; and (3) whether the levels of OXT in seminal EVs are associated with the fertility of AI boars.

**Methods:** Seminal EV samples were isolated by size-exclusion chromatography from entire ejaculates and ejaculate fractions: the first 10 mL of the sperm-rich fraction (SRF; SRF-P1), the remainder of the SRF (SRF-P2), and the post-SRF fraction. The OXT concentration was measured using a direct competitive immunoassay with AlphaLISA technology and an anti-OXT monoclonal antibody.

**Results:** Seminal EVs carry OXT, primarily on the outer surface of the EV membrane, likely within the protein corona. The concentration of OXT in seminal EVs varied among ejaculate fractions (*P* < 0.0001). It was higher in SRF-P2 (3,017.68 ± 860.52 pg/mL of SP) and post-SRF (3,613.27 ± 935.08 pg/mL of SP) EVs than in SRF-P1 (1,675.15 ± 520.62 pg/mL of SP) EVs. The concentration of OXT in seminal EVs was associated with the fertility outcomes of AI boars. Higher concentrations were found in the seminal EVs of boars with a high farrowing rate and in those with a small litter size.

**Conclusion:** Porcine seminal EVs carry OXT outside of membranes, and those originating from the accessory sex glands are particularly enriched. The association of OXT of seminal EVs with fertility is ambivalent: it enhances farrowing rate while potentially reducing litter size, likely via its effects on myometrial contractility, which facilitates sperm transport but may hinder embryo implantation.

## INTRODUCTION

Seminal plasma (SP) is the fluid surrounding spermatozoa during and after ejaculation, secreted mainly by the epididymis and accessory sex glands^[1]^. SP has a complex composition that includes active molecules which regulate sperm function^[1-3]^ and modulate the immune environment of the female reproductive tract to ensure optimal sperm performance and embryo development^[4,5]^. These active molecules include hormones such as oxytocin (OXT)^[6,7]^ and anti-Müllerian hormone^[8]^.

OXT is a peptide hormone primarily synthesized in the hypothalamus with a wide range of biological functions, including the ability to promote smooth muscle contractility^[9,10]^. In addition to the hypothalamus, OXT is synthesized in other body organs, including those of the male reproductive tract^[9]^. Measurable levels of OXT have been reported in SP of different mammalian species, including pigs^[7]^ and humans^[6]^. Seminal OXT has been positively associated with farrowing rates (FR) in sows^[7]^ and ewes^[11]^. This association has been linked to the putative role of seminal OXT in promoting myometrial smooth muscle contractility and, thereby, facilitating sperm transport after reaching the uterus following mating or artificial insemination (AI)^[12]^. However, OXT has a brief functional half-life of just a few minutes^[13]^, which challenges its proposed functional role in AI, as semen doses are often stored for hours or even days before use^[14]^. Seminal OXT would have a longer functional lifespan if it were protected from natural circulating inhibitors, such as aminopeptidases present in SP^[15]^. In this context, extracellular vesicles (EVs) could play a significant role, as the molecules they carry exhibit longer functional activity^[16]^.

EVs are nano-sized lipid bilayer membrane particles that are secreted by many functional cells and released into body fluids. They play a key role in cell-to-cell communication by transporting their cargo of active molecules from donor to recipient cells^[17]^. SP contains a greater number of EVs compared to other body fluids^[18]^. Seminal EVs can interact with and deliver their rich molecular cargo to sperm and functional cells of the female reproductive tract^[19]^. Therefore, they are involved in regulating some of the critical functional changes experienced by both spermatozoa and female reproductive tissues^[20,21]^. To our knowledge, no study has reported that seminal EVs, or EVs circulating in any other body fluids, carry hormones. However, the study by Bae & Watson suggests such a possibility because they reported the presence of OXT in secretory vesicles released from uterine epithelial cells in mares^[22]^. Based on this background, it can be hypothesized that EVs transport OXT. With this in mind, the primary objective of this study was to determine if seminal EVs carry OXT.

The study was conducted in pigs, one of the most important farm animal species, which is recognized as a valuable biomodel for human health^[23,24]^. Porcine ejaculate is expelled in fractions; therefore, in addition to EVs from the entire ejaculate, those from ejaculate fractions were analyzed to determine the origin of the seminal EVs carrying OXT. The ejaculate fractions included the first 10 mL of the so-called sperm-rich fraction (SRF), primarily containing SP from the epididymis (SRF-P1), the remainder of the SRF, primarily containing SP from the prostate (SRF-P2), and the sperm-poor or post-sperm fraction (post-SRF), primarily containing SP from the seminal vesicles^[25]^. As previously mentioned, higher levels of OXT in SP have been associated with increased FR in AI sows^[7]^. Therefore, if seminal EVs carry OXT, it is reasonable to hypothesize that the seminal OXT related to fertility would be transported by seminal EVs. Thus, the final objective of this study was to determine if OXT carried by seminal EVs is associated with the fertility of boars used in AI programs.

## METHODS

### Ethical statement

The animals were treated according to international guidelines on animal welfare (Directive 2010-63-EU) and the experiments were approved by the Bioethics Committee of the University of Murcia (Research Code CBE: 668/2025).

### Boars, ejaculates, and SP samples

The ejaculate donors were healthy, sexually mature Landrace and Large White boars included in AI programs. They were housed at an AI center owned by Artificial Insemination Management (AIM) Ibérica S.A. (Topigs Norsvin Spain SLU) in Calasparra, Murcia, Spain. This AI center complies with Spanish (ES300130640127, August 2006) and European (ES13RS04P, July 2012) regulations regarding animal health, semen collection, and marketing of AI doses.

The entire ejaculate was collected using a semi-automated method (Collectis®, IMV Technologies, L’Aigle, France), while the SRF-P1, SRF-P2, and post-SRF fractions were collected separately using the classic gloved hand method. Immediately after collection, the ejaculates underwent standard quality control procedures^[26]^. Only ejaculates that surpassed the thresholds set by the AI center for producing semen AI doses were chosen for experimentation. Specifically, the ejaculates had to contain more than 200 × 10^6^ spermatozoa/mL, with at least 70% motile spermatozoa and 75% morphologically normal spermatozoa.

The SP was collected immediately after ejaculation. Ten mL of semen samples from the entire ejaculate, SRF-P1, SRF-P2, and post-SRF were centrifuged twice at 1,500 g for 10 min at room temperature (RT) using a Rotofix 32A centrifuge (Hettich Centrifuge, Newport Pagnell, Buckinghamshire, England). Then, 8 mL of the supernatant from each sample was collected and treated with protease inhibitors (Roche Complete^TM^ Protease Inhibitor Cocktail Tablets, Basel, Switzerland). The SP samples were cooled to 5 °C and transported in thermoboxes to the Laboratory of the Animal Andrology Research Group at the University of Murcia (Campus de Espinardo, Murcia, Spain).

### Isolation of seminal EVs

Seminal EVs were isolated using the size exclusion chromatography (SEC) method described by Martínez-Díaz *et al*.^[27]^. Each SP sample was centrifuged twice: first at 3,200 g for 15 min at 4 °C using a Sorvall^TM^ STR40 centrifuge (Thermo Fisher Scientific, Waltham, MA, USA) and then at 20,000 g for 30 min at 4 °C using a Sorvall^TM^ Legend^TM^ Micro 21R centrifuge (Thermo Fisher Scientific). The resulting supernatant was used to isolate EVs. Prior to SEC, 2 mL of each supernatant was diluted in filtered phosphate buffered saline (PBS) (1:2, v/v), filtered with 0.22 μm Millex® syringe filters (Merck, Darmstadt, Germany), and concentrated using 100 kDa molecular weight cutoff Amicon® Ultra-4 mL centrifugal filters (Merck). The ultrafilters were spun at 3,200 g for 90 min at 4 °C. Homemade SEC columns were made using 20 mL filtration tubes (Econo-Pac® chromatography columns, Bio-Rad, Hercules, California, USA) and Sepharose CL-2B® (Merck). A total of 20 SEC fractions, each containing 500 μL, were collected, and fractions 7-10 were chosen as the richest in EVs. These fractions were mixed to generate a single 2 mL sample. The resulting seminal EV samples were divided into two aliquots. The first (500 μL) was stored at 5 °C for EV phenotypic characterization, and the second (1.5 mL) was stored at -80 °C until OXT analysis.

### Characterization of the extracellular vesicle samples

The seminal EV samples were phenotypically characterized according to the Minimal Information for Studies of Extracellular Vesicles 2023 (MISEV2023) guidelines^[28]^. Accordingly, the seminal EV samples were characterized using an orthogonal approach that included total protein concentration, particle concentration and size, EV morphology, protein EV markers, and the presence of non-vesicular extracellular particles (NVEPs).

The total protein concentration was measured using a NanoDrop^TM^ One spectrophotometer (Thermo Fisher Scientific). The spectrophotometer was set to measure protein absorption at 280 nm, and 2 µL of each seminal EV sample was used. Each seminal EV sample was analyzed in two technical replicates. The results were expressed in mg/mL. Particle concentration and size were evaluated using a ZetaView PMX-130 (Particle Metrix, Meerbusch, Germany). One µL of seminal EV sample diluted in Milli-Q water at a ratio of 1:2,000 (v/v) was used. The standard operating procedure “EV_488” in dispersion mode was used. Measurements were performed at a constant temperature of 24 °C and recorded using ZetaView software (version 8.04.02). Particle concentration was expressed as particles/mL of SP, and size was expressed in nanometers (nm). Each sample was analyzed twice. The morphology of seminal EVs was analyzed using cryogenic electron microscopy (cryo-EM). This analysis used a JEM-2200FS/CR field emission electron microscope (JEOL, Tokyo, Japan), which is equipped with a 200-kV field emission gun and a cooled ULTRASCAN 4000SP slow-scan camera with 4,008 pixels × 4,008 pixels (Gatan Inc., Pleasanton, California, USA). The analysis followed the protocol described in detail by Parra *et al*.^[29]^. The presence of the specific EV protein markers CD81 and HSP70, as well as albumin as an NVEP, was analyzed using a high-sensitivity flow cytometer (CytoFLEX S, Beckman Coulter, Brea, CA, USA). The cytometer was equipped with violet (405 nm), blue (488 nm), yellow (561 nm), and red (638 nm) lasers. The following antibodies were used: anti-CD81 (130-119-787, Miltenyi Biotec B.V. & Co. KG, Bergisch Gladbach, Germany); anti-HSP70/HSC70-APC (MA5-45093, Fisher Scientific); and anti-albumin-FITC (CLFAG16140, Cedarlane, Burlington, NC, USA). The analyses were performed in accordance with the recommendations of Welsh *et al*.^[30]^. This protocol involves using a recombinant green fluorescent protein (SAE0193, Merck) to verify the accuracy of the flow cytometer for EV input and counting, as well as using carboxyfluorescein succinimidyl ester (CFSE; CellTrace^TM^ CFSE, Thermo Fisher Scientific) to distinguish functional, intact EVs from NVEPs. Analyses were performed with a low flow rate of 5-10 μL/min, and at least 10,000 events were acquired per sample.

### Measurement of seminal OXT

The OXT was measured using a standardized and reliable direct competitive method^[31]^ that had been validated for SP samples^[7]^ and seminal EVs (unpublished results). The SP and seminal EV samples were diluted in AlphaLISA® Universal Buffer at ratios 1:64 and 1:4 (v/v), respectively. SP and seminal EV samples and OXT standards (Cusabio Technology LLC, Houston, TX, USA) were aliquoted at 15 µL in duplicate into 96-well plates. The standard curve consists of eight points which were generated from OXT conjugated to bovine serum albumin (from 0 to 2,400 pg/mL). The plates were then incubated for 90 min at RT in the dark with 15 μL of monoclonal anti-OXT antibody (30 μg/mL) coated with acceptor beads. The second incubation was performed by adding 10 μL of biotinylated OXT (1 nmol/L, Cusabio Technology LLC) to each well. The plates were then incubated at RT for 60 min. Finally, 10 μL of streptavidin-coated donor beads (20 μg/mL) were added to each well and incubated for 30 min at RT in the dark. Fluorescence intensity was measured using an EnSpire® Alpha Multimode Plate Reader (PerkinElmer, Waltham, MA, USA). Intra-assay and inter-assay variations were below 7.5% and 9.5%, respectively. OXT concentrations were expressed as pg per mL of SP.

### Experimental design

To avoid the effects of individual males and single ejaculates, the experiments were conducted using seminal EV samples isolated from SP mixtures. Each mixture came from several boars and ejaculates.

#### Experiment 1: Do seminal EVs carry OXT? If so, are they carried inside or outside?

**Figure 1:**
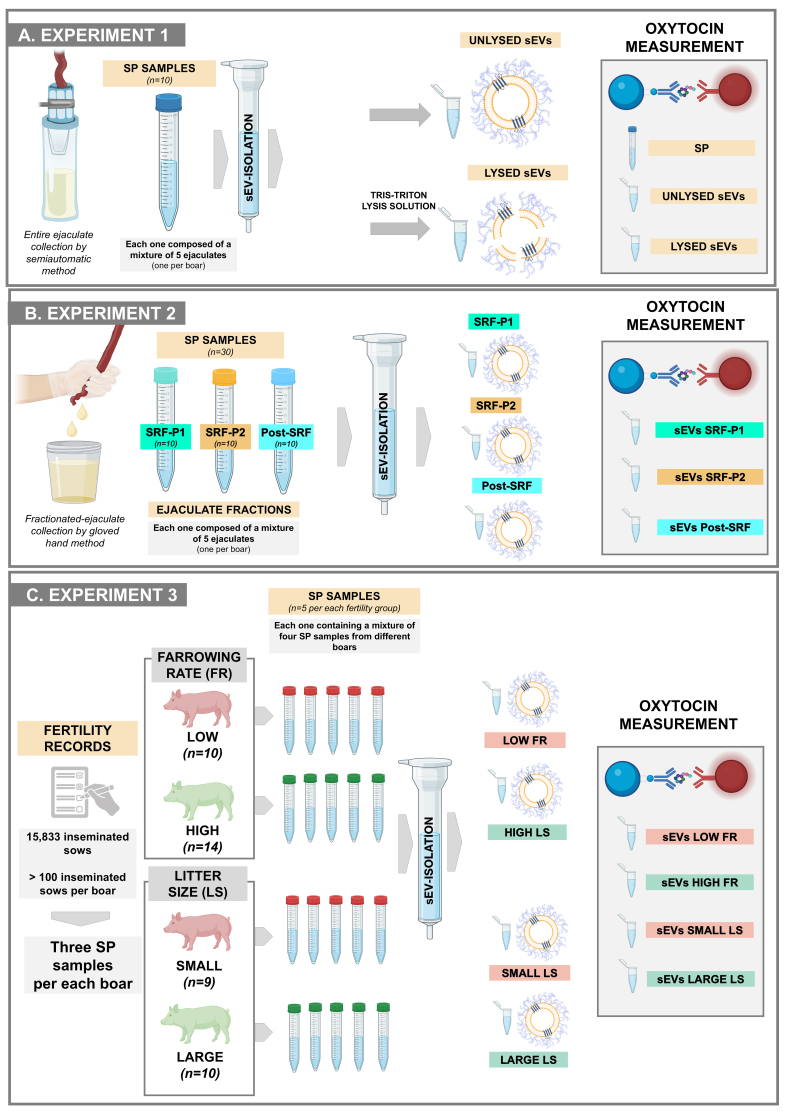
An overview of the experimental design adopted for each of the three experiments conducted. (A) Experiment 1 evaluated whether sEVs carried OXT and, if so, whether the OXT was carried inside or outside the sEVs; (B) Experiment 2 investigated whether the amount of OXT in sEVs differs between ejaculate fractions; (C) Experiment 3 investigated whether the concentration of OXT in seminal EVs was related to *in vivo* fertility outcomes in boars used in artificial insemination programs. Created in BioRender (Roca, J., 2025; https://BioRender.com/hqwnptw). Although some symbols were obtained from BioRender, Figure 1 was composed using Microsoft PowerPoint. SP: Seminal plasma; sEVs: seminal extracellular vesicles; SRF-P1: the first 10 mL of the sperm-rich fraction of the ejaculate (SRF); SRF-P2: the remainder of the SRF; Post-SRF: the ejaculate fraction that is poor in spermatozoa; FR: farrowing rate; LS: litter size.

This experiment used ten SP samples from the entire ejaculates [Figure 1A]. Each was harvested from a semen mixture of five entire ejaculates, each from a different boar. The concentration of OXT was quantified in the raw SP samples and in the isolated seminal EV samples. The seminal EV samples were treated with or without a lysis solution [50 mM Tris-HCl, 150 mM NaCl, 1% Triton X-100, 5 mM ethylenediaminetetraacetic acid (EDTA), at pH 7.4] to determine whether the OXT was inside the EVs (as cargo) or on the outer surface of the EV membrane. The OXT quantified in unlysed EV samples was considered to be on the outer surface of EVs. The OXT quantified in lysed EVs, minus the amount quantified in unlysed EVs, was considered to be inside the EVs. The OXT concentration was quantified in duplicate for each sample.

#### Experiment 2: Are there differences in the amount of OXT in the seminal EVs between ejaculate fractions?

Seminal EV samples were isolated from SP samples of the three different ejaculate fractions: SRF-P1, SRF-P2, and post-SRF [Figure 1B]. There were ten seminal EV samples from each ejaculate fraction. The SP samples used to isolate the seminal EVs came from a mixture of semen samples from five ejaculates collected from five different boars. The concentration of OXT was measured in duplicate for each sample.

#### Experiment 3: Is the concentration of seminal EV OXT related to in vivo fertility outcomes in boars used in AI programs?

The experiment used seminal EV samples isolated from the SP of 43 boars - semen donors in a commercial AI program [Figure 1C]. In this program, Landrace and Large White sows (*n* = 15,833) were inseminated twice cervically during postweaning estrus using 80 mL AI doses containing 2.4 × 10^9^ spermatozoa. The boars were selected because they inseminated more than 100 sows each, ranging from 113 to 706, and exhibited the greatest differences in FR and litter size (LS). FR is the percentage of sows that farrow relative to those inseminated, and LS is the number of piglets born per litter.

Boar fertility was recorded as the direct boar effect (DBE), which is the result of a statistical equation identifying the fertility variance attributed to the boar while excluding the variance attributed to other factors, such as farm, semen storage, or sow^[7]^. The DBE of each boar is expressed as the deviation in FR and LS, recorded as a percentage and number, respectively, relative to the average FR and LS of other boars of the same breed used in commercial AI programs at the same time. According to the DBE, the boars were divided into two groups. Boars with positive DBEs were considered to have high FR (*n* = 14) and large LS (*n* = 10) boars, while those with negative DBEs were considered to have low FR (*n* = 10) and small LS (*n* = 9). Seminal EVs were isolated from five SP mixtures in each fertility group (high FR and large LS, as well as low FR and small LS). Each SP mixture was created by randomly combining SP samples from three boars. OXT concentration was measured in duplicate in each seminal EV sample.

### Statistical analysis

The phenotypic characteristics of seminal EV samples and OXT concentration data were analyzed using the statistical program Prism 10 (GraphPad Software Inc., La Jolla, CA, USA). The Shapiro-Wilk test was used to determine whether the data followed a normal distribution. Accordingly, the data were analyzed using the *t*-test for paired data or the one-way analysis of variance (ANOVA) test with Tukey’s multiple comparison test. Differences were considered statistically significant at a *P*-value < 0.05.

## RESULTS

### Phenotypic characterization of seminal EV samples

**Figure 2:**
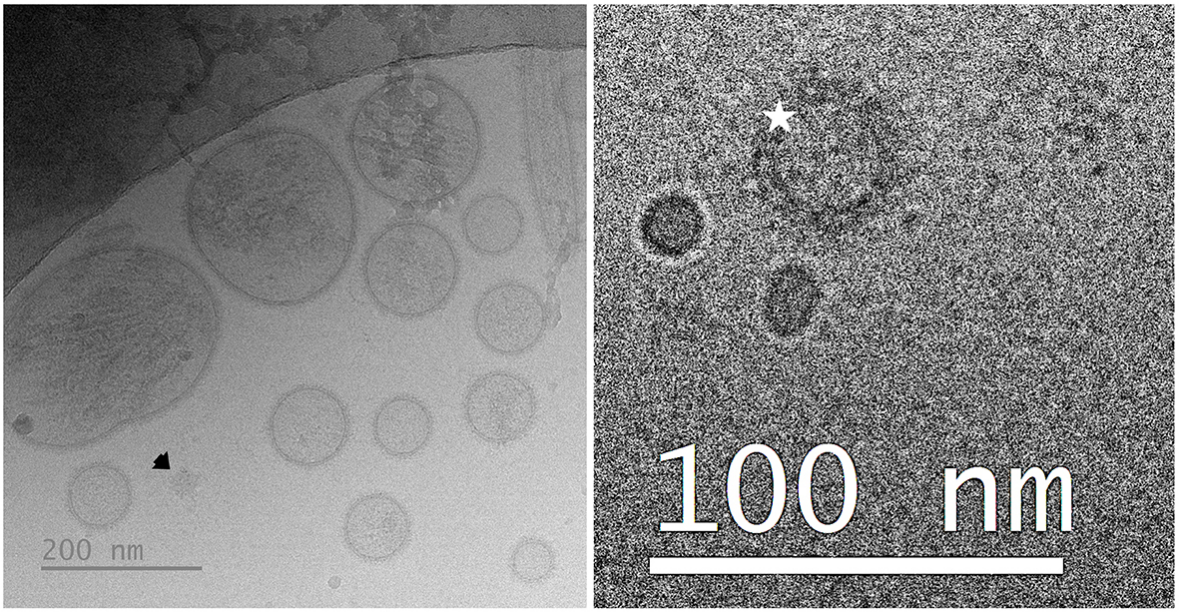
Representative cryogenic electron microscopy images of porcine seminal EVs. The left image shows a population of seminal EVs heterogeneous in size, shape, and electron density, with particles surrounded by a distinguishable membrane, as well as a non-vesicular extracellular particle (arrow). The right image shows a seminal EV surrounded by a protein corona (star). EVs: Extracellular vesicles.

Table 1 summarizes the phenotypic characteristics of the seminal EV samples used in each of the three experiments. All samples exhibited high particle concentrations, most of which were intact, functional seminal EVs (CFSE-positive particles). The low concentration of albumin indicates the high purity in all these samples. Notable results included the differences in particle concentration and size among the samples from the three ejaculate fractions (one-way ANOVA). The SRF-P1 and SRF-P2 samples had higher concentrations and smaller particle sizes than the post-SRF samples (*P* < 0.01; Tukey’s multiple comparison test). In Experiment 3, no differences were observed in any evaluated parameters between high- and low-FR samples. However, differences were noted in some parameters between large and small LS samples (*t*-test). Large LS samples had lower total protein and particle concentrations (*P* < 0.01 and *P* < 0.05, respectively), as well as higher percentages of CFSE-positive events (*P* < 0.01) and lower percentages of CD81-positive CFSE events (*P* < 0.05), compared to small LS samples. Despite these differences, a similar concentration of CFSE-positive particles was observed in seminal EV samples from large and small LS [mean ± standard deviation (SD): 143.22 × 10^9^ ± 44.11 × 10^9^ particles/mL in large LS and 207.28 × 10^9^ ± 36.01 × 10^9^ particles/mL in small LS]. The cryo-EM images showed that the seminal EVs were heterogeneous in terms of shape, size, and electron density, with a low presence of NVEPs [Figure 2].

**Table 1:** Phenotypic characteristics of the sEV samples in the three conducted experiments

**Exp**	**sEV** **source**	**Total protein** **(mg/mL)**	**NTA assessments**		**Flow cytometry analyses**			
			**Particle size** **(nm)**	**Particle concentration** **(1** × **10^9^ particles/mL)**	**CFSE+ events (%)**	**CFSE+ CD81+ (%)**	**CFSE+ HSP70+ (%)**	**Albumin (%)**
1	EE	0.55 ± 0.16	140.5 ± 7.9	261.1 ± 74.5	58.7 ± 8.4	65.5 ± 18.7	81.5 ± 11.8	6.3 ± 3.8
2	SRF-P1	0.29 ± 0.07	129.2 ± 4.8^x^	384.4 ± 88.6^x^	58.7 ± 8.3	58.6 ± 12.6	76.0 ± 7.5	6.8 ± 4.0
	SRF-P2	0.37 ± 0.21	138.6 ± 4.5^x^^,^^y^	362.5 ± 83.5^x^	64.7 ± 13.5	49.3 ± 8.1	79.1 ± 4.6	9.8 ± 3.9
	Post-SRF	0.51 ± 0.15	155 ± 15.2^y^	137 ± 4.6^y^	64.0 ± 11.5	46.6 ± 4.0	73.3 ± 14.8	11.6 ± 2.5
3	High FR	0.61 ± 0.15	142.0 ± 10.0	390.0 ± 101.3	53.9 ± 4.6	70.4 ± 12.0	84.2 ± 7.4	5.4 ± 1.3
	Low FR	0.46 ± 0.05	138.6 ± 5.2	387.5 ± 99.2	59.6 ± 4.4	82.3 ± 11.9	84.1 ± 11.3	4.9 ± 1.6
	Large LS	0.32 ± 0.09^x^	139.0 ± 10.2	295.7 ± 93.2^a^	60.7 ± 2.5^x^	51.1 ± 5.9^a^	80.1 ± 10.0	5.0 ± 0.7
	Small LS	0.69 ± 0.13^y^	142.1 ± 7.9	555.0 ± 123.3^b^	47.3 ± 4.4^y^	67.1 ± 8.7^b^	90.1 ± 5.4	6.3 ± 1.4

^a,b^*P* < 0.05; ^x,y^*P* < 0.01. sEV: Seminal plasma extracellular vesicle; Exp: experiment; EE: entire ejaculate; SRF-P1: first 10 mL of the SRF; SRF-P2: rest of the SRF; post-SRF: post sperm rich ejaculate fraction; SRF: sperm rich ejaculate fraction; FR: farrowing rate, LS: litter size; NTA: nanoparticle tracking analysis; CFSE: carboxyfluorescein succinimidyl ester; HSP70: heat stress protein 70.

### Experiment 1: Seminal EVs carry OXT on the outer surface of the EV membrane

**Figure 3:**
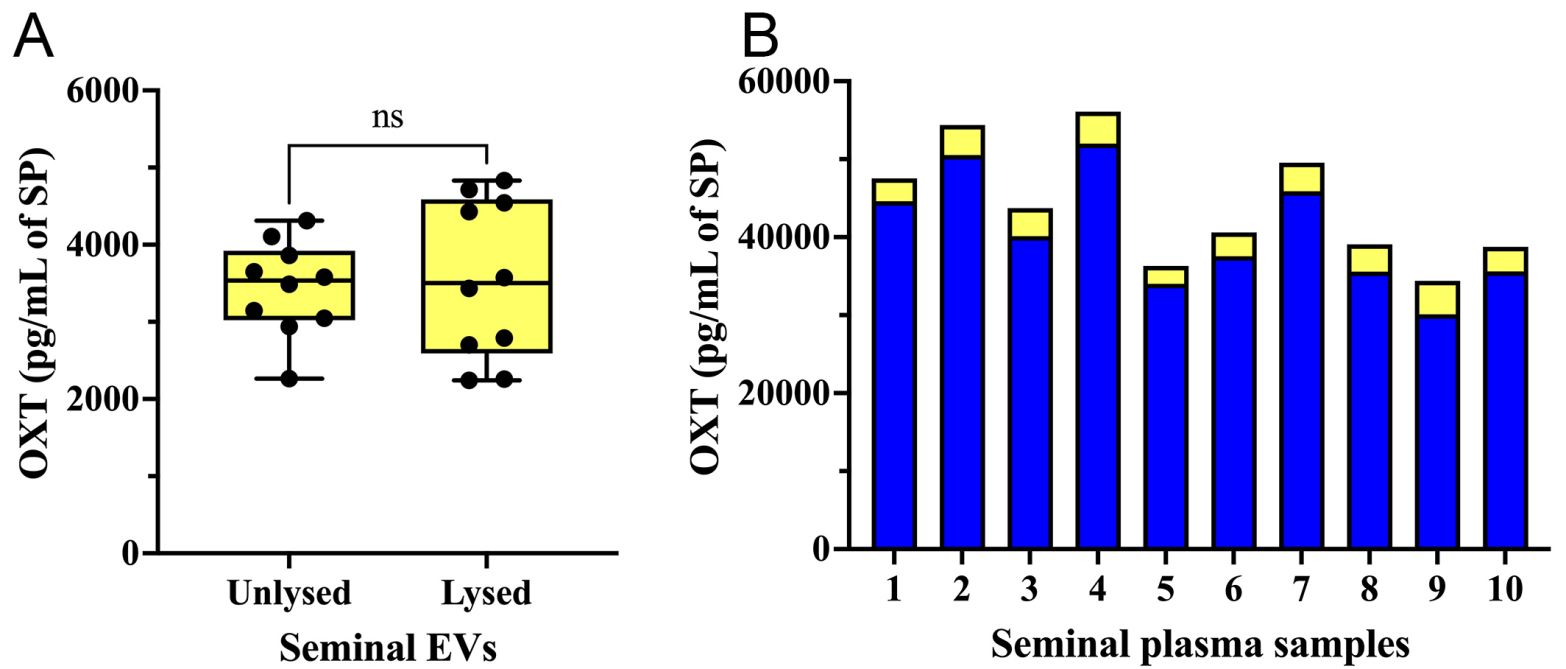
(A) OXT concentration (pg per mL of SP) in unlysed and lysed seminal extracellular vesicle (seminal EVs) samples; (B) OXT concentration in raw SP samples (blue bars) and in the seminal EVs isolated from the same SP samples (yellow bars). ns: No significant; SP: seminal plasma; OXT: oxytocin; EVs: extracellular vesicles.

The concentration of OXT was similar (*P* > 0.05; *t*-test) in both lysed and unlysed seminal EV samples [Figure 3A]. This indicates that OXT is present on the outer surface of the EV membrane. Seminal EV samples contained OXT at concentrations ranging from 2,264.08 pg/mL to 4,312.72 pg/mL (mean ± SD: 3,439.64 ± 607.62 pg/mL). These concentrations corresponded to 5.64%-10.72% of the total OXT amount measured in raw SP samples (mean ± SD: 40,606.01 ± 7,343.97 pg/mL, ranging from 30,096.04 to 51,993.50 pg/mL) [Figure 3B].

### Experiment 2: Seminal EVs from SRF-P2 and post-SRF are particularly enriched in OXT

**Figure 4:**
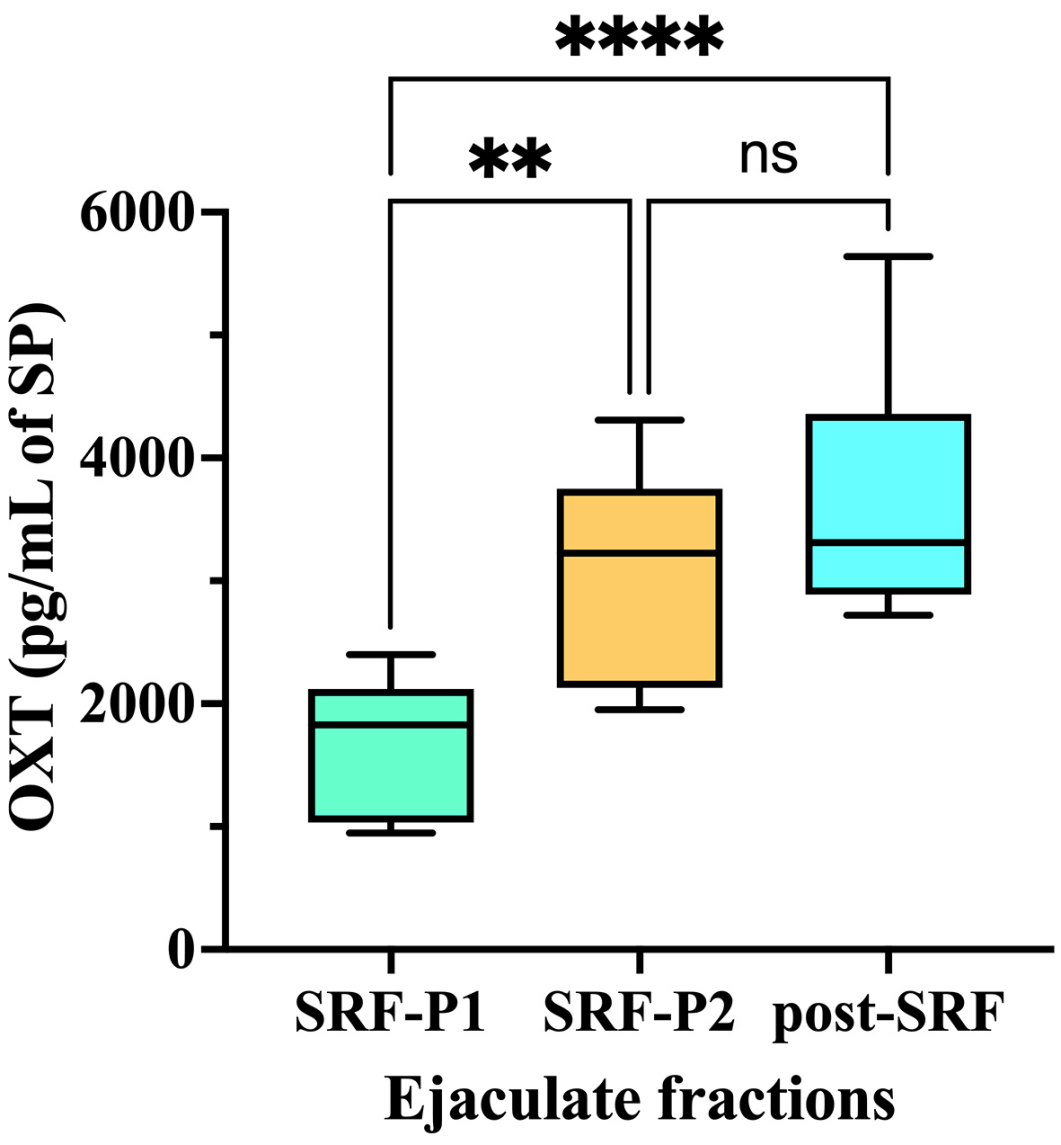
OXT concentration (pg per mL of SP) in seminal extracellular vesicles isolated from SP samples of three separate porcine ejaculate fractions: the SRF-P1, the SRF-P2, and the post-SRF. ^****^P < 0.0001; ^**^P < 0.01. ns: No significant; SP: seminal plasma; OXT: oxytocin; SRF-P1: first 10 mL of the sperm rich fraction; SRF-P2: remainder of the sperm rich fraction; post-SRF: post sperm rich fraction.

Seminal EV samples of the three ejaculate fractions contained OXT [Figure 4]. However, the concentration of OXT in the EVs differed among the ejaculate fractions (one-way ANOVA). The OXT concentration in SRF-P1 EVs was lower than in SRF-P2 and post-SRF EVs (*P* < 0.0001; Tukey’s multiple comparison test). These results suggest that seminal EVs secreted from the accessory sex glands are more enriched in OXT than those secreted from the epididymis.

### Experiment 3: The concentration of OXT was higher in seminal EVs of boars with high FR and small LS

**Figure 5:**
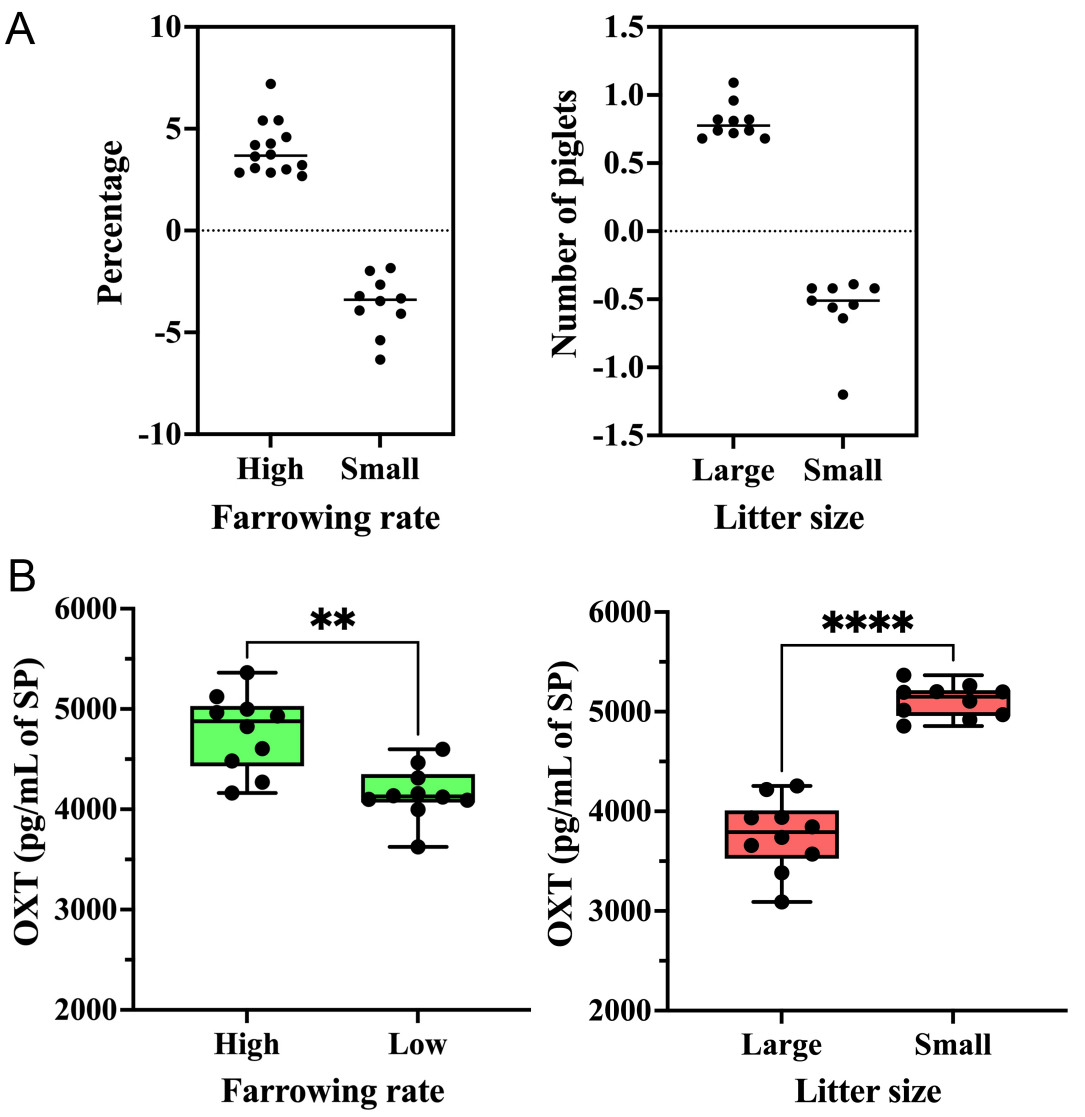
(A) The DBE of boars selected as seminal plasma donors on fertility experiment. The average DBE of all boars included in artificial insemination programs is represented by zero on the y-axis. The dots represent boars chosen for exhibiting the greatest differences in DBE for farrowing rate (right) and litter size (left); (B) OXT concentration (pg/mL of SP) in seminal extracellular vesicles of boars with high and low farrowing rates (right) and in seminal extracellular vesicles of boars with large and small litter sizes (left). ^****^P < 0.0001; ^**^P < 0.01. DBE: Direct boar effect; OXT: oxytocin; SP: seminal plasma.

The DBE (mean ± SD; range in parentheses) of boars with high FR was 4.01% ± 1.29% (from 7.20% to 2.68%), while the DBE of boars with low FR was -3.62% ± 1.41% (from -6.33% to -1.84%). For LS, boars with large LS had a DBE of 0.8 ± 0.13 piglets (from 0.68 to 1.09), while boars with small LS had a DBE of -0.57 ± 0.25 piglets (from -1.20 to -0.39) [Figure 5A].

The levels of OXT were measured in seminal EV samples isolated from boars with high and low FR and large and small LS. As shown in Figure 5B, seminal EV samples from boars with high FR had higher OXT concentrations (*P* < 0.01; *t*-test) than those from boars with low FR. Seminal EV samples from boars with large LS had lower OXT concentrations (*P* < 0.0001; *t*-test) than those from boars with small LS [Figure 5B].

## DISCUSSION

EVs play roles in various endocrine and metabolic disorders^[32,33]^. Accordingly, a recent scientific statement from the Endocrine Society encouraged the scientific community to identify the molecular cargo of EVs involved in endocrine pathways^[34]^. Our study aligns with this goal because it is, to our knowledge, the first demonstration that EVs carry functionally active hormones. Specifically, we show that porcine seminal EVs carry OXT, a small peptide hormone involved in both female and male reproductive physiology^[9,35]^. Numerous studies have examined the protein cargo of EVs^[36-39]^, including those from boar SP^[40]^; however, none have identified functional endocrine peptides or proteins. The failure to quantify OXT in the study of the porcine seminal EV proteome^[40]^ may be due to the limited sensitivity of the analytical instrument used. Despite improvements in the accuracy and sensitivity of the latest generations of mass spectrometry-based instruments, identifying and quantifying low-abundance proteins remains challenging.

The first experiment revealed that porcine seminal EVs contain OXT in varying amounts, representing five to ten percent of the total OXT in raw SP samples. Boar SP is particularly rich in OXT, and its concentrations can vary between and within boars^[7]^. The first experiment also showed that OXT is located outside the EV membrane. It is likely found in the protein corona surrounding the EV membrane externally. The protein corona is a dynamic and complex set of molecules, primarily proteins, that bind non-covalently to the outer surface of the EV membrane. This corona consists of two layers: an internal, permanent layer known as the hard layer and an external, transient layer known as the soft layer^[41]^. The composition of the soft layer shifts based on the molecular makeup of the environment encompassing the EVs^[42]^. Free OXT circulating in the SP is likely to become trapped in the soft layer of the protein corona of seminal EVs. The testes, epididymis, and accessory sex glands supply OXT to the SP^[43]^. It is important to note that molecules embedded in the protein corona are protected from natural inactivators, such as proteases or ribonucleases (RNases). This protection prolongs their functional half-life^[16,44]^. The protein corona is a defining feature of EVs, including seminal EVs^[29]^, as it plays an integral role in EV functionality. It affects EV kinetics, biodistribution, binding, and uptake by target cells^[42,44,45]^. OXT, when integrated into the EV protein corona, can bind to OXT receptors in the membrane of EV target cells. In the case of seminal EVs, these cells are spermatozoa and epithelial cells from the female genital tract.

The second experiment revealed differences in the concentration of OXT in seminal EVs (EV OXT) among ejaculate fractions, with those of SRF-P1 having the lowest concentration. These differences are likely related to variations in the amount of free OXT circulating in the SP of the ejaculate fractions. To our knowledge, no studies have evaluated OXT in SP from pig ejaculate fractions. However, it is known that the SP protein composition differs quantitatively rather than qualitatively among the three ejaculate fractions, probably due to their different origins^[15]^. Specifically, the SP of SRF-P1 primarily originates from the epididymis, the SP of SRF-P2 primarily originates from the prostate, and the SP of post-SRF primarily originates from the seminal vesicles^[25]^. Because SRF-P2 and post-SRF seminal EVs had the highest concentrations of OXT, it is reasonable to infer that the functional cells of the sex accessory glands secrete EVs with more OXT than the epididymis.

The third experiment revealed a relationship between seminal EV OXT concentrations and the fertility of boars used in an AI program. These results support our initial hypothesis that fertility-related SP OXT would be transported in EVs. However, the relationship was ambivalent: positive with FR and negative with LS. This finding is consistent, at least in part, with the observations of Padilla *et al*. who found a positive relationship between SP OXT levels and FR but not between SP OXT and LS^[7]^. Similarly, Okazaki *et al*. found that adding OXT to AI semen doses improved FR but not LS in inseminated sows^[46]^. Notably, the LS was smaller, though not significantly, in sows inseminated with OXT-containing AI doses in the study by Okazaki *et al*.^[46]^. The mechanical role that OXT plays in reproductive functions could explain the ambivalent action of seminal EV OXT on FR and LS. Sperm performance in the female reproductive tract influences FR more, while early embryo development and implantation influence LS more. Accordingly, seminal EV OXT could improve FR in two ways. First, it could improve sperm functionality and, consequently, its fertilizing capacity. Second, it could facilitate the travel of spermatozoa to the utero-tubal junction by activating myometrial contractions. The sperm plasma membrane expresses the OXT receptor, which is most prevalent in the flagellum region^[47]^. The expression of OXT preprotein messenger RNA (mRNA) in human spermatozoa has been found to be positively related to total and progressive motility^[43]^. Thus, Lymperi *et al*. suggested that SP OXT may be important for sperm motility^[43]^. Specifically, SP OXT regulates mitochondrial function in spermatozoa^[48]^. The endometrium and the myometrium of sows have OXT receptors^[49]^ and SP OXT facilitates sperm transport in the female genital tract by inducing myometrial contractions^[50,51]^. Therefore, it is reasonable to hypothesize that seminal EV OXT binds to the sperm membrane or myometrial receptors, thereby improving sperm motility and/or activating myometrial contractions. These effects would facilitate sperm transport to the utero-tubal junction and improve the ability of sperm to fertilize, consequently, increasing FR.

This study would be the first to demonstrate a negative association between OXT and LS. Previous studies have shown that OXT reduces embryo implantation rates^[52]^. These negative effects would be linked to the positive role of OXT in uterine contractile activity, which can hinder embryo implantation. Numerous studies have shown that treatment with an OXT antagonist improves embryo implantation, particularly in humans following embryo transfer^[53]^. Therefore, it can be hypothesized that OXT from seminal EVs stimulates myometrial OXT receptors in the early stages of pregnancy, thereby compromising embryo implantation. Notably, OXT receptors are present in the myometrium of sows during the early stages of pregnancy, including days 14-16, when embryo implantation occurs^[54]^. Seminal EVs are involved in embryo implantation by mechanisms that are not fully elucidated^[21]^.

The study identified a relationship between seminal EV OXT and male fertility. However, these findings should be considered preliminary because only five EV samples were analyzed per fertility group. Therefore, validation in future studies using larger boar cohorts is necessary. The study also proposes that seminal EV-derived OXT stimulates myometrial contractility, a hypothesis that requires confirmation in follow-up experiments.

In conclusion, this study demonstrated that porcine seminal EVs can carry OXT on the outer surface of their membranes, potentially within the protein corona. The concentration of EV OXT varied among ejaculate fractions, with the highest levels found in EVs from SRF-P2 and post-SRF, which originated mainly from the accessory sex glands. Seminal EV OXT showed an ambivalent relationship with the fertility of boars used in AI programs: it was positively associated with FR and negatively associated with LS. This ambivalence may reflect the proposed role of seminal EV OXT in stimulating myometrial contractility, which enhances sperm transport and fertilization but may impair embryo implantation. However, the ability of seminal EV OXT to induce myometrial contractions has not yet been demonstrated. Therefore, further experimental studies are required to confirm this.

## DECLARATIONS

### Availability of data and materials

The data and materials that support the findings of this study are available from the corresponding author upon reasonable request.

### Copyright

© The Author(s) 2025.
